# Changes in Cytokine, Filarial Antigen, and DNA Levels Associated With Adverse Events Following Treatment of Lymphatic Filariasis

**DOI:** 10.1093/infdis/jix578

**Published:** 2017-11-15

**Authors:** Britt J Andersen, Jessica Kumar, Kurt Curtis, Nelly Sanuku, Samson Satofan, Christopher L King, Peter U Fischer, Gary J Weil

**Affiliations:** 1 Infectious Diseases Division, Department of Medicine, Washington University School of Medicine, St Louis, Missouri; 2 Center for Global Health and Diseases, Case Western Reserve University School of Medicine, Cleveland, Ohio; 3 Papua New Guinea Institute of Medical Research, Goroka

**Keywords:** lymphatic filariasis, therapy, adverse events, cytokines, circulating filarial antigenemia

## Abstract

**Background:**

Mild to moderate adverse events (AEs) are common after treatment of lymphatic filariasis (LF) and pose a major challenge for the global LF elimination program. We studied changes in cytokine levels and filarial worm components in plasma of subjects with and without AEs following treatment of LF.

**Methods:**

Participants (n = 24) were hospitalized and monitored for AEs following treatment. Cytokines (27), filarial DNA, circulating filarial antigen (CFA), and immune complexes were measured in plasma samples collected before and after treatment.

**Results:**

Levels for 16 cytokines increased after treatment in individuals with moderate AEs compared to individuals with no and/or mild AEs. These included 3 major proinflammatory cytokines (interleukin 6, tumor necrosis factor α, and interleukin 1β). Eotaxin-1 levels were elevated at baseline in individuals who developed moderate AEs after treatment; thus, eotaxin-1 is a potential biomarker for AE risk. CFA and filarial DNA levels increased more in individuals with moderate AEs after treatment than in people with no/mild AEs.

**Conclusions:**

Increases in cytokine, filarial DNA, and CFA levels were associated with development of AEs following treatment of LF. Improved understanding of the pathogenesis of AEs may lead to improved methods for their prevention or management that could increase compliance in elimination programs.

## Teaser

Adverse events (AEs) are common after lymphatic filariasis treatment, and pose a major challenge for elimination programs. We identified cytokine profiles associated with posttreatment AEs, and showed that AEs were associated with increases in filarial DNA and antigen in plasma.

Lymphatic filariasis (LF) is a neglected tropical disease that is caused by the nematode parasites *Wuchereria bancrofti*, *Brugia malayi*, and *Brugia timori*. Adult worms release first-stage larvae (microfilariae [Mf]) into the blood, and these are ingested by mosquitoes. The parasites develop to become infective third-stage larvae that can initiate new infections when they are transmitted to humans by mosquitoes. The worms and the host’s inflammatory responses can lead to severe morbidity with lymphedema, hydrocele, and elephantiasis [1].

The World Health Organization (WHO) launched the Global Program to Eliminate LF (GPELF) in the year 2000 with the goal of eliminating LF as a public health problem by 2020. The primary tool used by GPELF is annual mass drug administration (MDA), and some 500 million people are treated each year [2]. The medications used—namely, albendazole (ALB), ivermectin (IVM), and diethylcarbamazine (DEC)—have well-established safety profiles, and serious adverse events (AEs) related to treatment are very rare. However, mild to moderate AEs such as fever and headache are common. High MDA compliance is important for LF elimination programs [3]. The fear of AEs in communities receiving MDA reduces compliance [4, 5]. Understanding the pathogenesis of AEs is even more urgent at this time, because recent studies have shown that a single dose of all 3 LF MDA drugs (IVM, DEC and ALB, sometimes called “IDA”) is more effective for clearing Mf than the current 2-drug MDA regimens [6] (authors’ unpublished observations). This increased efficacy may be associated with increased AE rates in infected individuals.

The pathogenesis of AEs after treatment of LF is poorly understood. Host immune responses and parasite death are believed to be involved, because posttreatment AE rates are much higher in infected individuals, and because AE rates are correlated with blood Mf counts [7]. AEs are probably related to release of parasite antigens and/or *Wolbachia* (an intracellular alpha proteobacteria found in LF-causing filarial worms). *Wolbachia*-derived molecules may interact directly with the innate immune system through ligands such as Toll-like receptors (TLRs) to activate immune cells to release cytokines [8–13]. Few studies have looked at changes in cytokines associated with AEs following LF treatment. One study documented increases in interleukin (IL) 6 and tumor necrosis factor alpha (TNF-α) after treatment in 5 men with AEs [14]. Another study reported that AEs were associated with increased levels of IL-6, lipopolysaccharide binding protein (LBP), IL-10, and soluble TNF receptor [15]. Other studies suggested that AEs may be related to circulating immune complexes (CICs) that form when filarial antigens are released by dying parasites [16, 17]. CICs from plasma of LF-infected individuals have been shown to be proinflammatory when added to granulocytes [18]. A recent clinical trial provided us with the opportunity to use 21st-century methods to revisit the issue of AE pathogenesis.

## METHODS

### Study Design

Plasma samples used for this study were obtained during a pharmacokinetic trial conducted in Papua New Guinea in 2013 [6]. Twenty-four *W. bancrofti*–infected individuals were randomized to 1 of 2 treatment arms: the standard LF MDA regimen for Papua New Guinea (ALB and DEC) or the new IDA triple therapy regimen. The AE assessment protocol was described in a previous publication [6]. In brief, objective AEs were assessed (vital signs and a brief physical examination) for all study participants at 0, 4, 8, 12, 24, 48, and 72 hours posttreatment in a hospital setting. Subjective AEs were assessed at the same times by asking the participants open-ended questions about symptoms that developed after treatment. All participants were followed as outpatients and examined on day 7. Nineteen of 24 participants (79%) developed at least 1 AE, and 7 of these individuals had fever >38°C. Blood was collected immediately before treatment and at 11 time-points after treatment (1–72 hours). Notable in this study was the high Mf levels (geometric mean, 1679 [range, 133–13776] Mf/mL). Plasma samples were stored and shipped at –80°C. Informed consent was obtained from all participants as previously described [6].

### Adverse Events Classification

AEs were scored as none, mild, or moderate. Those with moderate AEs (n = 7) had at least 1 new symptom plus objectively measured fever (a temperature of ≥38°C) within 72 hours after treatment. Individuals with subjective or objective AEs without fever were considered to have mild AEs (n = 12). Individuals with no objective or subjective symptoms were considered to have no AEs (n = 5).

### Cytokine Assay

Twenty-seven cytokines were measured using a MAGPIX system with the Bio-Plex Human 27-Plex Cytokine Panel and Bio-Plex Cytokine Reagent Kit (Bio-Rad, Hercules, California). Plasma samples were thawed and centrifuged before testing. A preliminary study tested samples from all 12 time-points (pretreatment and 1, 2, 3, 4, 6, 8, 12, 24, 36, 48, and 72 hours posttreatment) for 7 participants. Since there were no changes in cytokine levels during the first 6 hours, only 7 time points (pretreatment and 8, 12, 24, 36, 48, and 72 hours posttreatment) were tested for the remaining 17 study participants. All samples were tested in duplicate, and all samples from the same individual were run on the same plate. The cytokine assay panel included IL-1β, IL-1Ra, IL-2, IL-4, IL-5, IL-6, IL-7, IL-8, IL-9, IL-10, IL-12 (p70), IL-13, IL-15, IL-17, basic fibroblast growth factor (FGF), eotaxin-1, granulocyte colony-stimulating factor (G-CSF), granulocyte macrophage colony-stimulating factor (GM-CSF), interferon gamma (IFN-γ), interferon gamma-induced protein 10 (IP-10), monocyte chemoattractant protein 1 (MCP-1), macrophage inflammatory protein (MIP) 1β and 1α, platelet-derived growth factor BB (PDGF-BB), regulated on activation, normal T cell expressed and secreted (RANTES), TNF-α, and vascular endothelial growth factor (VEGF). Standard curves were calculated using the manufacturer’s software, and our analysis considered mean concentrations (picograms per milliliter) from 2 duplicate wells. Mean levels for all 27 cytokines were calculated for each AE group at each time-point. Kruskal–Wallis *H* tests were used to compare absolute cytokine levels between the 3 AE groups at each time-point. Wilcoxon signed-rank tests were used to compare posttreatment levels to baseline levels within AE groups for each time-point. After evaluation of the data, 3 outliers with extremely elevated levels at baseline were excluded from the analysis.

### Circulating Filarial Antigen

A direct sandwich enzyme immunoassay was performed as previously described [19, 20]. Plasma samples were available for 21 of the 24 individuals for this test (6 moderate, 10 mild, and 5 no AEs), and samples from 5 time-points were tested (pretreatment and 6, 12, 24, and 48 hours posttreatment). All samples from individual participants were tested in duplicate on the same plate. The mean circulating filarial antigen (CFA) levels were calculated for each AE group at each time-point. Kruskal–Wallis *H* tests were used to compare the absolute CFA levels between the 3 AE groups at each time-point. Wilcoxon signed-rank tests were used to compare posttreatment CFA levels at each time-point to baseline levels within the AE groups.

### Detection of Filarial DNA by Quantitative Polymerase Chain Reaction

DNA was extracted from 100 µL of plasma using the E.Z.N.A. Tissue DNA Kit (Omega Bio-tek) using the manufacturer’s protocol. The quantitative polymerase chain reaction (qPCR) assay was a TaqMan probe-based assay, and the target was the “long DNA repeat” of *W. bancrofti* (GenBank accession number AY297458). We used previously published primers and probes [21] purchased from Integrated DNA Technologies. Real-time PCR reactions were performed with 10 μL of TaqMan master mix (Applied Biosystems) plus 450 nmol/L of primers, 125 nmol/L probe, and 2 μL DNA with a final volume of 20 μL. Thermal cycling was performed with a QuantStudio 7-Plex Real-Time PCR System (Applied Biosystems). PCR reactions were carried out for 40 cycles, and cycle threshold (Ct) values were determined using the manufacturer’s software. Plasma samples were available for 21 of the 24 individuals for this assay (7 moderate, 9 mild, and 5 no AEs), and 7 time-points were selected (pretreatment and 8, 12, 24, 36, 48, and 72 hours posttreatment). Samples were run in duplicate, and all samples from individual participants were tested on the same plate. Each plate contained a positive control (DNA extracted from *W. bancrofti* Mf), and 2 negative controls (DNA extracted from plasma samples from healthy North American control subjects and deionized water). Delta Ct values (baseline Ct value minus posttreatment Ct value) were calculated at each time-point for each individual, and 1-way analysis of variance analysis was used to compare the ∆Ct values between the 3 AE groups at each time-point.

### Immune Complex Assay

In this assay, CICs were incubated with human C1q (part of the first component in the classical complement pathway) that was immobilized on microtiter plates. C1q was purchased from Sigma-Aldrich. Nunc Immulon 2HB flat-bottom 96-well plates (Thermo Scientific) were coated with 50 µL of 0.01 mg/mL C1q in 1× phosphate-buffered saline (PBS) pH7.4 and incubated at 4°C overnight. Plates were washed and blocked for 1 hour at room temperature. After washing, 50 µL sample plasma or standard (diluted 1:60 in PBS with 0.5% casein, 0.5% Tween-20) was added to each well, and the plates were incubated at room temperature for 1 hour. Aggregated human γ-globulin (AHG) was used as the positive control and standard. Alkaline phosphatase-conjugated goat antihuman immunoglobulin G was used at a dilution of 1:1000, and plates were incubated for 1 hour at 37°C. The plates were developed with alkaline phosphatase substrate (pNPP disodium salt hexahydrate) and read at 405 nm. Plasma was available from 22 of the 24 individuals for this assay (7 moderate, 10 mild, and 5 no AEs), and 7 time-points were selected (pretreatment and 8, 12, 24, 36, 48, and 72 hours posttreatment). Samples were run in duplicate and all samples from individual participants were run on the same plate. Each plate contained 2 negative controls (plasma samples from healthy North American control subjects and deionized water). Values were expressed as nanograms per milliliter of AHG, and mean CIC values were calculated for each AE group at each time-point. Kruskal–Wallis *H* tests were used to compare absolute CIC levels between the 3 AE groups at each time-point. Wilcoxon signed-rank tests were used to compare posttreatment CIC levels at each time-point to baseline levels within each AE group.

## RESULTS

### Cytokine Levels

Three of the 24 individuals (all in the mild AE group) had extremely elevated cytokine levels at baseline, and they were excluded from the analysis. These 3 individuals had outlier levels for 9, 12, and 14 of the 27 measured cytokines respectively, at baseline. These high baseline cytokine levels were unrelated to treatment and their inclusion would have distorted the data, because the aim of the study was to investigate the change in cytokines posttreatment.

**Figure 1.**
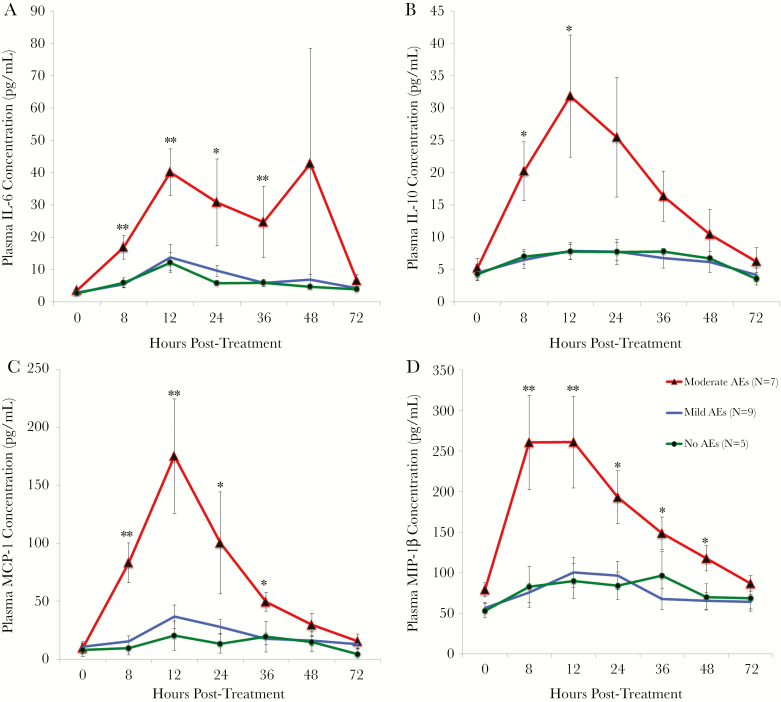
Mean cytokine levels (± standard error [SE]) in the 3 adverse event (AE) groups over time. Interleukin 6 (IL-6) (*A*), interleukin 10 (IL-10) (*B*), monocyte chemoattractant protein 1 (MCP-1) (*C*), and macrophage inflammatory protein 1β (MIP-1β) (*D*) increased posttreatment in the moderate AE group, whereas there were no significant changes in the no or mild AE groups. Significance (Kruskal–Wallis *H* test): * *P* < .05; **P < .01.

**Figure 2.**
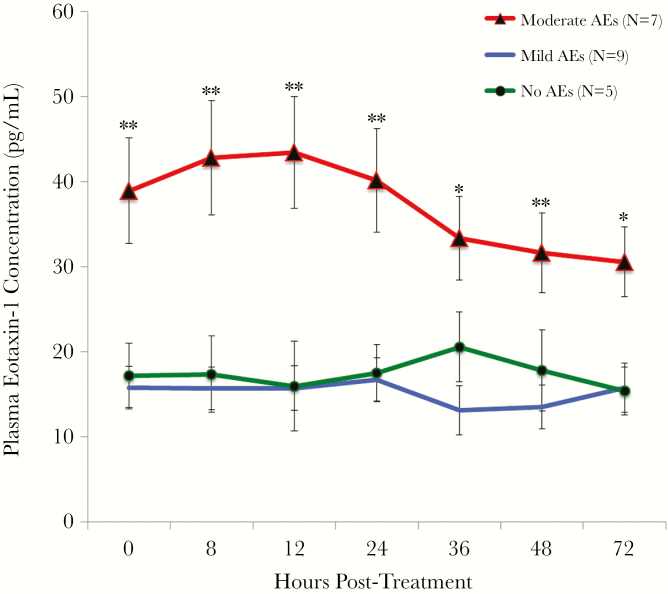
Mean eotaxin-1 levels (± standard error) for all 3 adverse event (AE) groups over time. Eotaxin-1 levels were significantly higher at all time-points in the moderate AE group compared with the no and/or mild AE groups. Significance (Kruskal–Wallis *H* test): *P < .05; **P < .01.

Changes in cytokine levels after treatment were significantly different in persons with moderate AEs compared to those in persons with no and/or mild AEs for 22 of the 27 cytokines tested (Table 1) (Kruskal–Wallis analysis followed by post hoc tests to determine which AE groups were significantly different). Most of these cytokines (IL-1β, IL-1Ra, IL-4, IL-6, IL-7, IL-10, IL-12, IL-17, G-CSF, IP-10, MCP-1, MIP-1α, PDGF-BB, MIP-1β, TNF-α, and VEGF) increased significantly more in the moderate AE group after treatment compared to the no and/or mild AE groups. IL-6, IL-10, MCP-1 and MIP-1β had the most dramatic posttreatment increases in the moderate AE group (Figure 1). Several cytokines (IL-8, IL-13, and eotaxin-1) were significantly higher at baseline in persons who developed moderate AEs. Eotaxin-1 was greatly increased pretreatment in 6 out of the 7 individuals who later developed moderate AEs (Figure 2). Baseline eotaxin-1 levels were not correlated with baseline Mf counts. The finding that some cytokines were higher at baseline in individuals who would go on to develop AEs was surprising, and we decided to redo the analysis including the 3 excluded individuals. There was still a significant difference in baseline eotaxin-1 and IL-8 levels between individuals with moderate AEs compared with individuals with no and/or mild AEs. However, the difference in IL-13 at baseline disappeared when the outliers were included.

**Table 1.** Changes in Cytokines at Different Times After Treatment in Persons Who Developed Moderate Adverse Events (AEs) After Treatment of Filariasis Compared to Those With No or mild AEs

Hours post treatment	IL-1β	IL-1Ra	IL-2	IL-4	IL-6	IL-7	IL-8	IL-10	IL-12	IL-13	IL-15
0							+			+	
8	++	++		+	++		++	+	+	++	-
12	++	++		+	++		+	+		+	
24		+	-	+	+		+			+	
36	+	+			++		++			+	-
48						+					-
72											
Hours post treatment	IL-17A	Eotaxin-1	G-CSF	IP-10	MCP-1	MIP-1α	PDGF-BB	MIP-1β	RANTES	TNF-α	VEGF
0		++							- -		
8	+	++	+	+	++	++		++	-	++	
12		++	++	++	++	++	+	++	-		+
24		++		++	+			+	-	+	
36		+	+	++	+			+		+	
48		++		++				+			
72		+		+							

These differences were due to significant increases (represented with plus signs) in cytokine levels in the moderate AE group compared to the no and/or mild AE groups (Kruskal-Wallis analysis followed by post-hoc tests to determine which AE groups were statistically different). IL-2, IL-15 and RANTES were exceptions as these cytokines were decreased (represented with minus signs) before and/or after treatment in the moderate AE group. Significance (by the Kruskal-Wallis H Test): + or - corresponds to *P* < .05; ++ or - - corresponds to *P* < .01.

Values for several cytokines were lower at baseline (RANTES) or decreased significantly posttreatment (IL-2 and IL-15) in people who developed moderate AEs compared with people with no and/or mild AEs. Five cytokines (IL-5, IL-9, basic FGF, GM-CSF, and IFN- γ) did not differ by AE group at any time-point; however, IL-5 did increase posttreatment as previously described [22, 23]. We did not correct for multiple comparisons, so additional data would be needed to confirm our findings. However, the differences in cytokine levels were dramatic between the AE groups, and they persisted over time making the results more credible. Based on the standard significance level of .05, approximately 3 differences would be expected to be significant by chance for 54 tests (27 cytokines measured at 2 time-points). We found 28 significant differences in cytokines levels at 12 and 24 hours posttreatment.

### CFA Levels and Mf Counts

**Figure 3.**
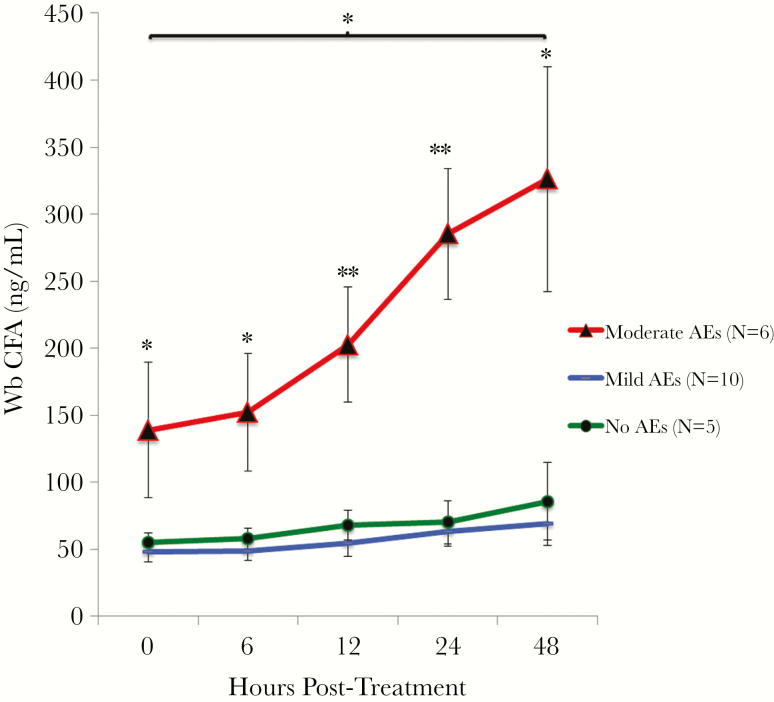
Mean *W. bancrofti* (Wb) circulating filarial antigen (CFA) levels (± standard error) for each adverse event (AE) group over time. CFA levels were significantly higher in the moderate AE group than in the no and mild AE groups. Significance (Kruskal–Wallis *H* test): *P < .05; **P < .01. CFA levels were significantly higher at 48 hours posttreatment compared with baseline in the moderate AE group (*P* = .048 by Wilcoxon signed-rank test).

CFA is known to circulate in the blood of LF-infected individuals, and this antigen is used as a diagnostic marker. The detection limit of the CFA enzyme immunoassay assay is 6.3 ng/mL. CFA was detected in all samples from all study subjects. Baseline CFA levels were positively correlated with baseline Mf counts (Spearman ρ = 0.66, *P* = .001), and absolute CFA levels were significantly higher at baseline in the individuals who later developed moderate AEs. CFA levels were significantly higher in the moderate AE group compared to the no/mild AE groups at all time-points (Figure 3). CFA levels increased in all groups after treatment, but the difference was only significant at the 48-hour time-point (*P* = .048 by Wilcoxon signed-rank test) compared to the baseline level in the moderate AE group. There was no difference in absolute CFA levels between the 2 treatment arms at any time-point.

Baseline Mf counts were higher in individuals who developed moderate AEs (geometric mean, 4491 Mf/mL vs 1111 Mf/mL in the mild AE group and 1351 Mf/mL in the no AEs group), and this difference was significant (*P* = .017 by the Kruskal–Wallis test).

### Filarial DNA Levels

**Figure 4.**
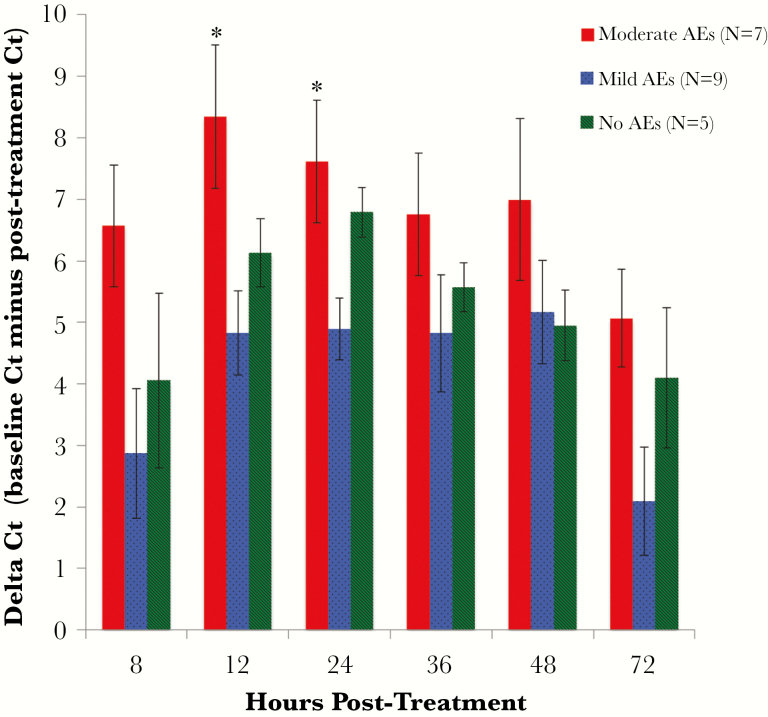
Filarial DNA levels in plasma (expressed as ∆ cycle threshold [Ct] ± standard error) after treatment by adverse event (AE) group. Ct values decreased after treatment in all 3 AE groups, signifying an increase in filarial DNA levels in plasma. Mean ∆Ct values were significantly greater in the moderate AE group compared with the mild AE group at 12 (*P* = .025) and 24 (*P* = .020) hours posttreatment (by analysis of variance). *P < .05.

Thirty-three percent of participants had detectable filarial DNA in plasma collected before treatment. There was no correlation between pretreatment Ct values and baseline Mf counts (Spearman ρ = –0.1). Filarial DNA was detected in plasma of all subjects at 8 hours posttreatment. Also, filarial DNA levels increased (Ct values decreased) after treatment in persons with filarial DNA detected at baseline. DNA levels quickly increased for the first 12–24 hours after treatment, after which they start to decrease. However, filarial DNA was still detectable in plasma in 95% of individuals 72 hours posttreatment. There was a statistically significant difference in ∆Ct values between the 3 AE groups at 12 and 24 hours posttreatment, and this difference was due to significantly higher ∆Ct values (larger increase in DNA levels) in the moderate AE group compared to the mild AE group (Figure 4). A similar trend was observed between the moderate and no AE groups, but the difference was not statistically significant due to the small number of individuals in the no AE group (n = 5). There were no significant differences in filarial DNA levels in plasma by treatment arm at any time-point.

### CIC Levels

CIC were detected in all samples, and the range at baseline was 108–1312 ng AHG equivalent/mL (median, 417 ng). Baseline CIC levels were positively correlated with Mf counts (Spearman ρ = 0.68, *P* = .006). There was no difference in absolute CIC levels between the AE groups at any time-point (Supplementary Table 1). CIC levels were relatively stable after treatment, and there were no consistent patterns as differences included both increases and decreases.

## DISCUSSION

The aim of this study was to determine the role of host cytokines, and filarial components released by parasites after treatment, in the development of AEs in LF-infected individuals. This is the most detailed study to date of cytokine responses that occur in persons with AEs after treatment of LF. We identified 22 cytokines that were differentially regulated posttreatment between the 3 AE groups. The majority of these cytokines increased more posttreatment in persons who developed moderate AEs. Our data are consistent with previously published results such as increases in IL-6, TNF-α, and IL-10 posttreatment in people with AEs [14, 15]. However, we also found that many other cytokines increased in participants with moderate AEs.

Plasma levels of filarial DNA and CFA both increased after treatment, and posttreatment increases in CFA were much more dramatic in people who developed moderate AEs than in those with no/mild AEs. Treatment with ALB/DEC and IVM/DEC/ALB resulted in similar changes in CFA and filarial DNA levels after treatment, suggesting that the 2 treatments have similar activity against Mf and adult filarial worms in the first days after treatment. CFA levels increased slightly later after treatment than filarial DNA, but the CFA increases persisted for a longer time. This result is consistent with prior reports of increased CFA levels 5–7 days posttreatment [24, 25]. Taken together, our results suggest that cytokines, filarial DNA, and CFA all may be involved in the pathogenesis of AEs. It is likely that the cytokine responses are triggered by molecules that are released from dying parasites. However, it is unclear whether cytokine release is triggered by phagocytosis of parasite debris or by direct interaction of parasite molecules with ligands on the surface of host cells. Compared to filarial DNA and CFA levels, CIC levels were stable after treatment, and there was no difference in CIC levels between the AE groups at any time-point. These results suggest that CIC may not be involved in the pathogenesis of moderate AEs.

Cytokine changes have been extensively studied in patients with septicemia or after exposure to endotoxin with sequential increases in TNF-α, IL-1β, IL-6, IL-1Ra, and IL-10 [26]. The cytokine pattern in this study was somewhat similar in that TNF-α was the first to increase with a peak at 8 hours posttreatment. IL-6 had the largest increase (a 13-fold increase in the moderate AE group) with a peak at 12 hours. IL-10, which increases later after endotoxin exposure, was not delayed in this study; it rose by 8 hours and peaked at 12 hours. Increases in proinflammatory cytokines may be stimulated by release of *Wolbachia* from dying filarial worms, but additional studies will have to be conducted to test this hypothesis. Dramatic increases in MCP-1 and MIP-1β/1α in persons with moderate AEs suggest that monocytes and/or macrophages are involved in the pathogenesis of AEs. Both of these cytokines are released by macrophages after endotoxin exposure [27], so this finding is consistent with the *Wolbachia* release hypothesis. Although *Wolbachia* do not contain lipopolysaccharide, they do contain endotoxin-like lipoproteins that interact with cellular ligands TLR2 and TLR6 [9, 11, 13].

Several baseline characteristics are known to be correlated with development of AEs after treatment of LF. For example, high Mf counts are a known risk factor [7], and our study confirmed that this is the case. High CFA levels have not been previously identified as a risk factor for AEs, perhaps because antigen levels were not measured. However, the association is not surprising, because CFA levels are positively correlated with Mf counts. The finding that participants with elevated levels of certain cytokines prior to treatment were at increased risk for moderate AEs was not anticipated. This was especially true for eotaxin-1, a chemokine secreted by various cells that attracts circulating eosinophils to their respective tissue [28]. One potential explanation for this finding is that individuals with high levels of eotaxin-1 may have activated eosinophils that are poised for attack. These cells rapidly kill parasites that have been damaged by anthelmintic drugs, and this may trigger a more vigorous proinflammatory response that results in AEs. This hypothesis is supported by the finding that eotaxin-1–deficient mice have reduced eosinophil responses to TLR2 activation and filarial antigen exposure, and the finding that macrophages from these mice produce less IL-6 [29]. Additional research will be needed to understand the apparent link between eotaxin-1 and AEs.

Samples from the Papua New Guinea trial were ideal for our study because of the high rate of AEs, the detailed clinical information available for participants, and the availability of plasma at many time-points after treatment. One limitation is that we were unable to assess *Wolbachia* in the plasma samples for technical reasons. Another limitation is that the correlations we observed do not prove causation.

In conclusion, this study has provided additional information on changes in plasma cytokine levels and in filarial worm components that are associated with moderate AEs after LF treatment. We have also shown that high Mf counts and high levels of CFA and eotaxin-1 at baseline are associated with increased risk for moderate AEs. Taken together, our results suggest that components released from filarial worms interact with the host immune system to release proinflammatory cytokines that lead to moderate AEs with fever and associated symptoms. More work is needed to identify the specific filarial worm components that are responsible for this immune activation, but *Wolbachia* endobacteria represent an attractive candidate. However, they are unlikely to be the only contributor, because similar AEs occur in patients after treatment of loiasis, and *Loa loa* does not contain *Wolbachia* [30]. Improved understanding of the pathogenesis of AEs may lead to strategies to prevent or manage AEs in ways that increase compliance with MDA, which is essential for the success of LF elimination programs.

## Supplementary Data

Supplementary materials are available at *The Journal of Infectious Diseases* online. Consisting of data provided by the authors to benefit the reader, the posted materials are not copyedited and are the sole responsibility of the authors, so questions or comments should be addressed to the corresponding author.

Supplemental Table

## Notes

***Acknowledgments.*** The authors thank the technicians of Maprik District, Papua New Guinea, for their support during sample collection.

***Financial support.*** This work (sample collection) was supported by the Bill & Melinda Gates Foundation (grant number GH5342) and by a grant from the Barnes-Jewish Hospital Foundation.

***Potential conflicts of interest.*** All authors: No reported conflicts of interest. All authors have submitted the ICMJE Form for Disclosure of Potential Conflicts of Interest. Conflicts that the editors consider relevant to the content of the manuscript have been disclosed.

Presented in part: 65th Annual Meeting of the American Society of Tropical Medicine and Hygiene, Atlanta, Georgia, 13–17 November 2016. Scientific Session 166, Presentation 1874.
