# Supplementary material for: Changes in Cytokine, Filarial Antigen, and DNA Levels Associated With Adverse Events Following Treatment of Lymphatic Filariasis
Source: J Infect Dis. 2017 Nov 15;217(2):280–7. doi: 10.1093/infdis/jix578 (PMC5853815; doi:10.1093/infdis/jix578)
Supplement: Supplemental Table [file jix578suppl_supplemental_table.docx]

Supplemental Table 1: Levels of circulating immune complexes (CIC) in plasma at different times after treatment in the three adverse events (AEs) groups.

| Hours Post-Treatment | 0 | 8 | 12 | 24 | 36 | 48 | 72 |
| --- | --- | --- | --- | --- | --- | --- | --- |
| Moderate AEs  (N=7) | 514 (144) | 512 (168) | 539 (146) | 510 (158) | 514 (95) | 587 (207) | 534 (162) |
| Mild AEs (N=10) | 373 (96) | 384 (96) | 378 (90) | 391 (90) | 386 (102) | 361 (207) | 396 (110) |
| No AEs  (N=5) | 611 (175) | 615 (129) | 635 (154) | 699 (139) | 702 (143) | 698 (154) | 648 (151) |

Mean CIC plasma concentration, ng aggregated human immunoglobulin equivalent/mL (+ SE).
